# Furmonertinib in lung adenocarcinoma with EGFR exon 20 insertion mutation: A case report of positive outcome

**DOI:** 10.1097/MD.0000000000046998

**Published:** 2026-01-23

**Authors:** Qilin Wang, Beilei Zeng, Chuanyu You, Tian Xu, Xinyu Chen, Xueping Yang, Juncai Ye, Yan Gui

**Affiliations:** a Department of Oncology, Affiliated Hospital of North Sichuan Medical College, Nanchong, Sichuan Province, China; b School of Medical Imaging, North Sichuan Medical College, Nanchong, Sichuan Province, China.

**Keywords:** EGFR exon 20 insertion, furmonertinib, lung adenocarcinoma

## Abstract

**Rationale:**

epidermal growth factor receptor (EGFR) mutations play a pivotal role in non-small cell lung cancer (NSCLC), with the EGFR exon 20 insertion (EGFR20ins) mutation being the third most prevalent alteration in the EGFR gene. Patients harboring this mutation typically exhibit a greater tumor burden and poorer overall prognosis. This mutation is strongly associated with resistance to EGFR-TKIs, presenting significant challenges for clinical management. Furmonertinib, an innovative third-generation EGFR-TKI, has been validated in numerous trials for its effectiveness in targeting the EGFR20ins mutation. Against this background, the study aims to explore the clinical application of furmonertinib in patients with advanced EGFR20ins-mutant NSCLC.

**Patient concerns:**

A patient with advanced EGFR20ins-mutant NSCLC initially presented with a significant tumor burden, resulting in rapid disease progression and a decline in quality of life, which subsequently led to a poor prognosis.

**Diagnoses:**

Next-generation sequencing of the tumor tissue identified an EGFR20ins mutation (p.p772_H773insPHP) with a mutation prevalence of 83.58%.

**Interventions:**

The patient received high-dose furmonertinib as first-line treatment. Throughout the treatment, tumor burden, disease stability, progression-free survival (PFS), and adverse effects were closely monitored.

**Outcomes:**

The patient experienced prolonged advantages from high-dose furmonertinib as initial therapy and attained a PFS of 27 months. The patient demonstrated substantial reduction in tumor burden, attained extended disease stability, and noted enhancements in overall quality of life, accompanied by tolerable adverse effects.

**Lessons:**

Furmonertinib, as a third-generation EGFR-TKI, demonstrated substantial efficacy in patients with advanced EGFR20ins-mutant NSCLC, significantly extending PFS and improving quality of life. This drug offers a promising new treatment option for patients with resistance to conventional therapies. Further studies will help validate the application of furmonertinib in a broader patient population.

## 1. Introduction

Research data reveal that lung cancer is the most prevalent cancer globally and the primary cause of cancer-related mortality.^[1]^ Non-small cell lung cancer (NSCLC) constitutes over 85% of all instances, with lung adenocarcinoma as the predominant subtype.^[2]^ In Chinese lung adenocarcinoma patients, the epidermal growth factor receptor (EGFR) mutation rate is 50.2%, predominantly characterized by exon 19 deletions at 24.3%, followed by the L858R point mutation in exon 21 at 22.9%.^[3]^ In the past ten years, the advancement of targeted treatments, including tyrosine kinase inhibitors (TKIs), has transformed therapy alternatives for EGFR-positive patients with NSCLC, especially those with susceptible mutations in exons 19 and 21. The effectiveness of EGFR-TKIs varies considerably for uncommon EGFR mutations beyond the prevalent sensitive variants. The EGFR exon 20 insertion (EGFR20ins) mutation constitutes 10% of all EGFR-positive NSCLC cases and is distinguished by resistance to first- and second-generation EGFR-TKIs.^[4]^ In 2021, mobocertinib and amivantamab received approval for administration in NSCLC patients exhibiting the EGFR20ins mutation. Nonetheless, the effectiveness of these medications has been inadequate.^[5,6]^ We present a case of a lung adenocarcinoma patient with an EGFR20ins mutation (p.p772_H773insPHP), who experienced prolonged benefits from furmonertinib as first-line therapy, attaining a progression-free survival (PFS) of 27 months and remains under observation, a result not previously attained.

## 2. Case description

**Figure 1.**
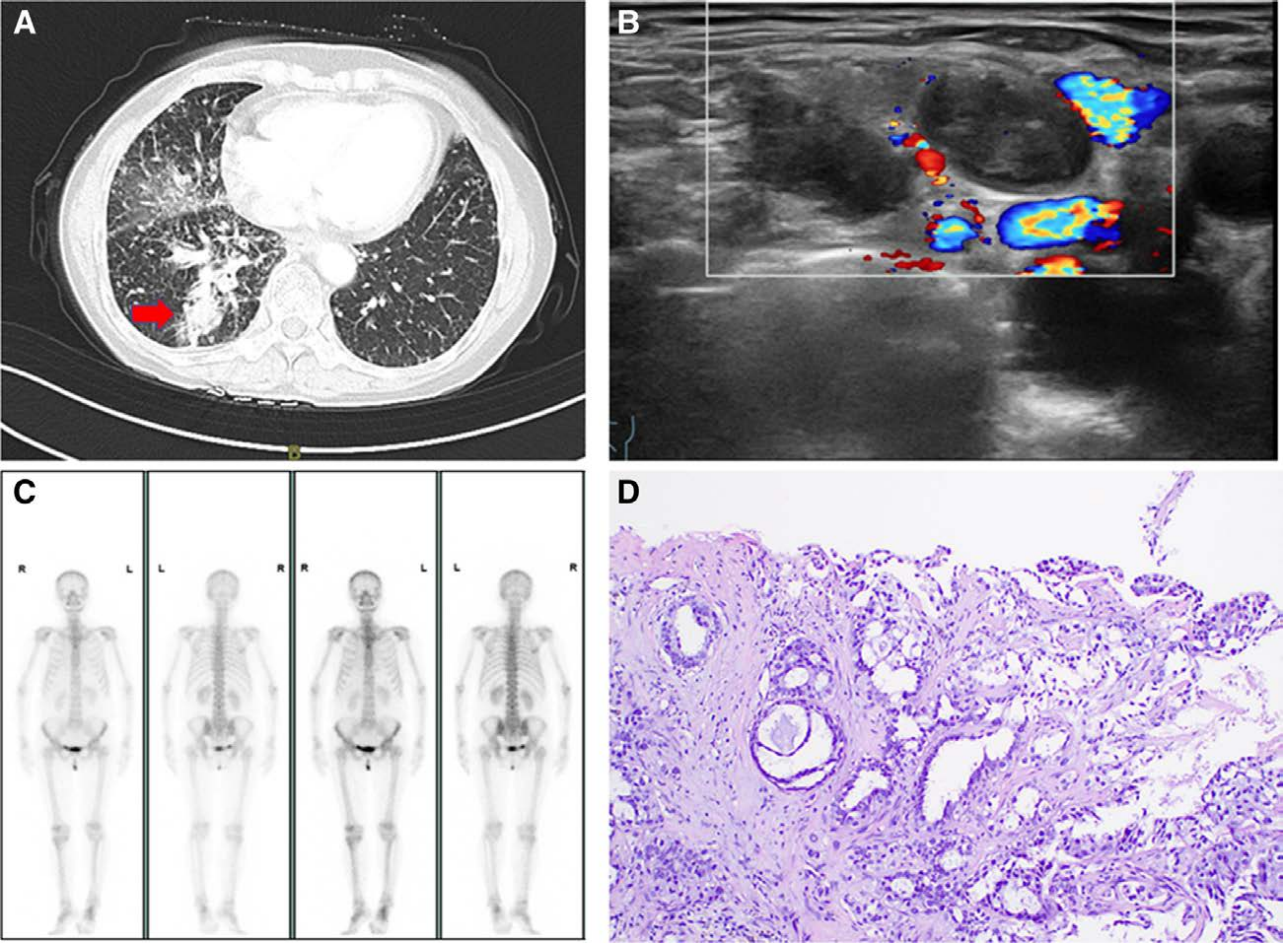
Dynamic changes in tumor during treatment and baseline date of the patient. (A) CT Image. The CT scan shows a 2.5 cm × 1.8 cm mass in the posterior basal segment of the lower lobe of the right lung. (B) Ultrasound of the cervical lymph nodes. The ultrasound reveals enlargement of the right supraclavicular and cervical lymph nodes. (C) SPECT-CT Image. SPECT-CT indicates no bone metastasis. (D) Representative histopathological image of the tumor (H&E staining). CT = computed tomography, H&E = hematoxylin and eosin, SPECT-CT = single photon emission computed tomography.

**Figure 2.**
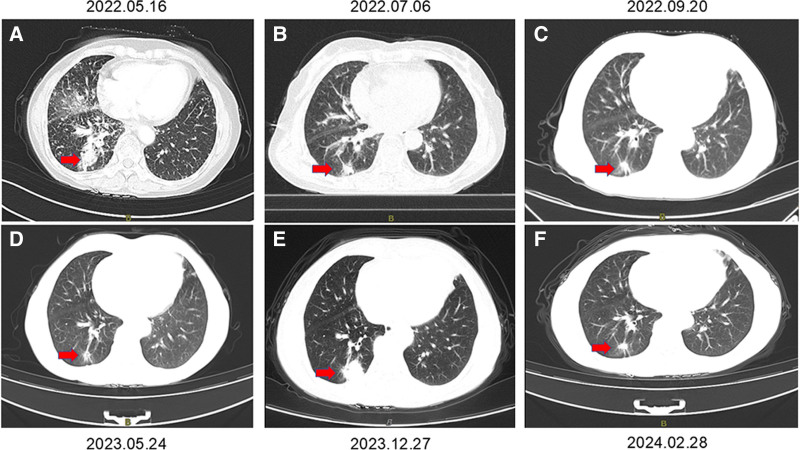
Representative CT Images at Different Time Points. The CT scan shows a lesion in the lower right lung, with the lesion indicated by a red arrow. CT = computed tomography.

The patient, a 56-year-old female, arrived to our hospital on May 16, 2022, with a 1-month history of cough and phlegm production. The patient has a history of hypertension, with no prior smoking or alcohol intake. A chest CT identified a mass in the posterior basal region of the right lower lobe (2.5 cm × 1.8 cm) exhibiting cavitation, along with disseminated nodules in both lungs and enlarged mediastinal and bilateral hilar lymph nodes, indicating a tumor with multiple pulmonary metastatic lesions (Fig. 1A and 2A). Neck ultrasound revealed enlarged lymph nodes in the right supraclavicular fossa and level 4 of the neck, measuring 1.5 cm × 0.9 cm (Fig. 1B). The brain MRI, whole-body bone scan, and stomach and adrenal ultrasound showed no notable abnormalities (Fig. 1C). A CT-guided lung biopsy was conducted on May 19, 2022, following the exclusion of contraindications. The postoperative pathological analysis revealed modest epithelial cell dysplasia, focal coagulative necrosis, and dispersed atypical cells, indicative of adenocarcinoma. A biopsy of the right level 4 cervical lymph node indicated that the tumor in the right lung was adenocarcinoma (Fig. 1D). Next-generation sequencing of the tumor tissue identified an EGFR20ins mutation (p.p772_H773insPHP) with a mutation prevalence of 83.58%. PD-L1 tumor proportion score was 0%, and combined positive score was likewise 0%. The TP53 p.Q192 mutation was detected, demonstrating a mutation prevalence of 67.31%. The patient was diagnosed with stage IVa lung adenocarcinoma (T4N3M1a) based on these results.

**Figure 3.**
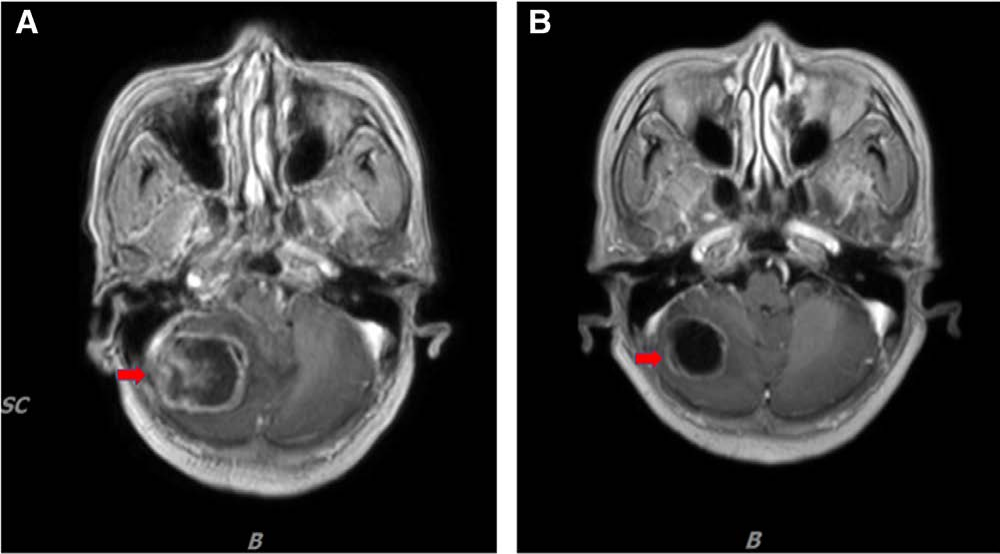
MRI images. Pre- and post-radiotherapy MRI scans reveal brain metastasis, with the lesion indicated by a red arrow. MRI = magnetic resonance imaging.

Following deliberation, oral furmonertinib at a dosage of 160 mg daily was commenced treating the patient in May 2022. On July 6, 2022, the patient had the initial follow-up following the commencement of therapy. No treatment-related adverse effects (TRAEs) were detected. Imaging revealed a notable decrease in the initial mass located in the posterior basal region of the right lower lobe (1.5 cm × 1.2 cm) and a reduction in mediastinal lymph nodes (Fig. 2B). Per the response evaluation criteria in solid tumors criteria, the therapeutic response was classified as partial remission (PR). The patient thereafter maintained consistent oral furmonertinib therapy. Subsequent CT scans on September 20 and November 30, 2022, demonstrated a notable decrease in the mass located in the posterior basal portion of the right lower lobe (1.3 cm × 0.6 cm) (Fig. 2C). Throughout the treatment, the patient manifested a minor rash and mouth ulcers. The treatment response was PR. Subsequent CT scans in May and September 2023 revealed a minor additional decrease in the mass located in the posterior basal region of the right lower lobe (0.8 cm × 0.6 cm) (Fig. 2D). No TRAEs were observed during the treatment, and the outcome was PR. A further CT scan in December 2023 indicated minor growth of the mass in the posterior basal portion of the right lower lobe (2.4 cm × 1.9 cm) (Fig. 2E). No TRAEs were observed, and the illness status remained stable (SD). The patient persisted with oral furmonertinib therapy. Subsequent CT scans in February and June 2024 indicated a decrease in the size of the mass located in the posterior basal portion of the right lower lobe (1.4 cm × 1.0 cm) (Fig. 2F). No TRAEs were observed, and the outcome was PR. In October 2024, the patient exhibited headache symptoms, and a brain MRI identified a right cerebellar metastasis measuring 3.5 cm × 3.8 cm × 3.0 cm (Fig. 3A). The therapy response indicated disease progression (PD). On October 25, 2024, stereotactic radiation (30 Gy in 5 fractions) commenced for the brain metastases, in conjunction with furmonertinib targeted therapy. In December 2024, the patient had a follow-up MRI that revealed a decrease in the brain metastasis (2.6 cm × 2.4 cm × 2.3 cm) (Fig. 3B). The patient has been administered high-dose furmonertinib (160 mg/day) as first-line treatment since May 2022, and as of the last update of this article (April 2025), the patient continues to derive benefits from furmonertinib, achieving an PFS of 27 months. No problems or significant adverse effects were seen with furmonertinib administration. The patient indicated that, despite encountering adverse effects such as dermatitis and mouth ulcers, her general quality of life has enhanced due to the treatment’s success, and she maintains optimism regarding potential future therapeutic results.

## 3. Discussion

The EGFR20ins mutation entails the insertion of 1 to 4 amino acids subsequent to the C-helix, facilitating the activation of the EGFR kinase domain. It constitutes 80% to 90% of all exon 20 insertions and is frequently correlated with EGFR amplification and TP53 mutations.^[7]^ TP53 mutations correlate with worse PFS during chemotherapy (pCHT) and EGFR-TKI therapy, although no significant association exists between the exon 20 insertion site and PFS or overall survival (OS).^[8]^ First-generation EGFR-TKIs are reversible ATP-competitive inhibitors, and the majority of cancers exhibiting exon 19 deletions or L858R EGFR activating mutations demonstrate sensitivity to these agents. Nevertheless, these medications exhibit minimal efficacy against EGFR20ins mutations. Until recently, chemotherapy was the conventional first-line treatment. A retrospective analysis indicated that advanced NSCLC patients with EGFR20ins mutations exhibited an overall response rate (ORR) of 19.2% and a disease control rate (DCR) of 41.3% at 6 months following first-line platinum-based chemotherapy, with a median PFS of 6.4 months.^[9]^ Mobocertinib and amivantamab have received approval for administration in advanced NSCLC patients exhibiting EGFR20ins mutations. The PFS for these treatments is 7.3 months and 8.3 months, respectively, with median overall survival (mOS) of 24.0 months and 22.8 months, and ORR of 28% and 40%, respectively. In a Phase III clinical trial evaluating first-line mobocertinib against platinum-based chemotherapy, mobocertinib failed to show a substantial advantage over chemotherapy. The occurrence of grade ≥ 3 TRAEs was greater in the mobocertinib cohort than in the chemotherapy cohort (62% vs 53%). The predominant TRAEs linked to amivantamab included grade 1 to 2 rashes (86%), infusion-related responses (66%), and paronychia (45%), with 4% of patients ceasing treatment.^[5,6]^ Nonetheless, their efficacy is subpar compared to that of EGFR-TKIs aimed at conventional EGFR mutations.

Furmonertinib, an osimitinib derivative, received approval in China in March 2021 for the treatment of NSCLC patients who acquire the EGFR T790M mutation during or subsequent to EGFR-TKI therapy.^[10]^ Research indicates that, in contrast to first-generation EGFR-TKIs, furmonertinib enhances the therapeutic window related to EGFR-WT and has relatively low susceptibility to EGFR-T790M resistance as an EGFR-TKI.^[11]^ Furmonertinib is predominantly metabolized by CYP3A4 and eliminated through feces. In human plasma, more than 95% of furmonertinib and its metabolites are covalently attached to plasma proteins, with the unbound drug primarily comprising the parent molecule and AST5902. Upon oral treatment, furmonertinib is fully absorbed and swiftly disseminated to tissues, with the highest concentration observed in the target organ, the lungs. Furthermore, both furmonertinib and its principal metabolite, AST5902, possess the ability to traverse the blood-brain barrier, offering enhanced optimism for the management of patients with brain metastases.^[12]^

In the Phase I dose-escalation study of furmonertinib, daily doses from 20 mg to 240 mg did not produce dose-limiting toxicities, and the maximum tolerated dose was not attained. In the Phase IIA dose-expansion study, administration of 160 mg daily vemurafenib in patients with brain metastases resulted in a central nervous system ORR of 84.6%, whereas the ORR for the 80 mg daily dosage was 60%. The elevated 160 mg dosage did not markedly augment the occurrence of severe adverse events relative to the 80 mg dosage.^[13]^ A 2024 study indicated that furmonertinib 240 mg shown significant treatment efficacy in patients with leptomeningeal metastases, yielding a median OS of 8.43 months and a clinical response rate of 75%. Only 6.3% of patients encountered grade 3 adverse effects, indicating that this dosage may establish the standard for treating brain metastases in EGFR-mutant NSCLC.^[14]^

The integration of targeted therapy with radiotherapy amplifies the inhibitory mechanisms on cellular survival pathways, hence augmenting the body’s ability to eradicate malignant cells. TKI medication obstructs the EGFR pathway, thereby inhibiting the downstream PIK3/AKT and RAS/RAF/MEK/ERK signaling cascades. This inhibition obstructs DNA damage repair, facilitates apoptosis, and diminishes cell growth.^[15]^ Radiotherapy temporarily compromises the integrity of the blood-brain barrier, enhancing its permeability and allowing the ingress of EGFR-TKIs into the central nervous system. This procedure elevates the medication concentration in brain metastases, hence augmenting its inhibitory effects on intracranial malignancies. Research has shown that the integration of TKI therapy with irradiation lowers the quantity of cells in the radiation-resistant S phase, thereby enhancing radiosensitivity and producing a synergistic effect.^[16]^

Numerous clinical studies have demonstrated the effectiveness of high-dose furmonertinib in managing advanced NSCLC patients with EGFR 20ins mutations. The most recent findings from the FAVOUR project were given verbally at WCLC 2023: Furmonertinib 240 mg daily was administered for the treatment of EGFR 20ins-mutant advanced NSCLC, demonstrating an ORR of 78.6%, a DCR of 100%, and a duration of response (DoR) of 15.2 months.^[17]^ The efficacy was markedly superior than conventional platinum-based doublet chemotherapy. Jia et al documented a case of an advanced NSCLC patient with an EGFR20ins mutation who experienced a favorable outcome from high-dose furmonertinib (160 mg/day), attaining a PFS of 10 months, without any significan TRAEs during the treatment duration.^[18]^ Chen et al documented a case involving an advanced NSCLC patient with an EGFR20ins mutation who underwent combination therapy with furmonertinib and anlotinib, resulting in sustained disease stability (SD) and favorable treatment tolerance.^[19]^ The FURTHER/FURMO-002 (NCT03846203), NCTO5466149, and FURVENT/FURMO-004 (NCT05607550) investigations are presently evaluating the efficacy of furmonertinib in larger patient cohorts with EGFR20ins mutations in NSCLC. These trials are anticipated to provide more insights into the therapeutic potential of furmonertinib.

Previous studies have reported 7 cases of patients with EGFR exon 20 insertion mutations treated with furmonertinib.^[18–23]^ The majority of these cases involved male patients, with a mPFS of 9 months (Table 1). In our case, the PFS was 27 months, exceeding the reported PFS of mobocertinib (7.3 months) and amivantamab (8.3 months). Moreover, the TRAEs were negligible, exhibiting only mild side effects, unlike the more prevalent and severe adverse events linked to mobocertinib. The 27-month PFS is a significant outcome; nonetheless, it is derived from a solitary patient, necessitating additional research with bigger cohorts to validate the generalizability of these results.

**Table 1:** Summary of cases of EGFR exon 20 insertion mutation patients treated with furmonertinib.

Author	Year	Age/gender	Treatment	PFS
Jia et al^[18]^	2022	58/M	High-dose furmonertinib (160 mg/d)	9 mo
Zhang et al^[20]^	2022	57/ M	High-dose furmonertinib (160 mg/d)	10 mo
Chen et al^[19]^	2023	68/ M	High-dose furmonertinib (160 mg/d) + anlotinib (12 mg/d)	11 mo
Han et al^[21]^	2023	38/F	High-dose furmonertinib (240 mg/d)	8.13 mo
Han et al^[21]^	2023	40/ M	High-dose furmonertinib (240 mg/d)	10.9 mo
Jiang et al^[22]^	2023	71/ M	high-dose furmonertinib (160–240 mg/d)	5 mo
Li et al^[23]^	2024	54/ M	Regular-dose furmonertinib (80 mg/d)	11 mo
Present case	2025	56/F	High-dose furmonertinib (160 mg/d)	27 mo

F = female, M = male, PFS = progression-free survival.

Resistance mechanisms to third-generation EGFR-TKIs can be classified into on-target resistance, off-target resistance, phenotypic transformation, and unspecified causes.^[24]^ On-target resistance arises from acquired mutations in the EGFR pathway, while off-target resistance is characterized by the activation of other signaling pathways. Phenotypic transformation denotes the conversion from adenocarcinoma to squamous cell carcinoma, small cell lung cancer, or other morphologically different neoplasms. The C797S mutation is the predominant resistance mechanism to furmonertinib, causing structural modifications in the EGFR protein that hinder medication binding. Additional pathways, including MET amplification, HER2 amplification, activation of the PI3K/AKT/mTOR pathway, and epithelial-mesenchymal transition, may possibly play a role in resistance, although the precise processes are still being explored.^[24]^

While medicines like osimertinib demonstrate significant efficacy in patients with traditional EGFR mutations (exons 19 and 21), their effectiveness is constrained in malignancies with the EGFR20ins mutation.^[25]^ Conversely, furmonertinib exhibits significant efficacy in patients with EGFR20ins mutations, rendering it a more promising therapeutic alternative for this particular mutation. The data from this singular case report are constrained, necessitating additional investigations with a larger cohort of EGFR20ins-mutant patients undergoing furmonertinib treatment to assess its efficacy and safety. Furthermore, investigating combination therapies or sequential treatments with alternative medications may yield synergistic results, especially in instances of extensive metastases or resistance to monotherapy. Recent investigations reveal that Sunvozertinib exhibits an ORR of 61% and a DCR of 88% in patients with EGFR20ins mutations, but additional results remain preliminary.^[26]^ The phase III PAPILLON study indicated that the combination of evorimab and chemotherapy achieved a mPFS of 11.4 months, markedly surpassing the chemotherapy-only group, which recorded a mPFS of 6.7 months, reflecting a 60% decrease in the risk of disease progression or mortality. The ORR in the evorimab plus chemotherapy combination group was 73%, markedly above the 47% recorded in the chemotherapy-only group.^[27]^ According to the PAPILLON research, evorimab received official approval from the National Medical Products Administration on February 11, 2025, for use in conjunction with carboplatin and pemetrexed as a first-line therapy for adult patients with locally progressed or metastatic EGFR20ins NSCLC. Clinical trials for pharmaceuticals like Zipalertinib (CLN-081), BEBT-109, PLB1004, JMT-101, BLU-451, FWD1509 MsOH, and YK-0229A are currently in progress, with expectations for the forthcoming outcomes.

Despite the aforementioned treatments exhibiting encouraging advancements, EGFR20ins mutations displ ay significant variability, with distinct subtypes revealing diverse responses to therapy. Patients may acquire resistance to targeted medicines, requiring the investigation of novel therapeutic techniques. The effectiveness of immune checkpoint drugs in patients with EGFR20ins mutations is currently constrained, necessitating additional investigation. Future investigations may concentrate on formulating targeted medicines for particular mutation subtypes and examining the integration of immunotherapy with targeted therapeutics.

## 4. Conclusion

This report evaluates the clinical efficacy and safety of first-line treatment with 160 mg daily of furmonertinib in a single patient with advanced NSCLC harboring an EGFR 20ins mutation, resulting in a PFS of 27 months. Throughout the treatment, the patient exhibited just a slight rash and mouth ulcers, with no TRAEs documented. As of the most recent follow-up in April 2025, the patient remains responsive to high-dose furmonertinib. Furmonertinib has demonstrated significant success in treating EGFR20ins NSCLC, offering a potential novel therapeutic alternative for this patient demographic. Nonetheless, further extensive investigations are required to validate its effectiveness in these patients.

## Acknowledgments

The authors thank the patients for their participation in this study.

## Author contributions

**Data curation:** Qilin Wang, Tian Xu, Xinyu Chen, Juncai Ye.

**Funding acquisition:** Beilei Zeng, Yan Gui.

**Investigation:** Qilin Wang, Beilei Zeng, Xueping Yang.

**Methodology:** Chuanyu You, Tian Xu, Xinyu Chen, Xueping Yang.

**Supervision:** Chuanyu You, Tian Xu, Xinyu Chen, Juncai Ye, Yan Gui.

**Writing – original draft:** Qilin Wang, Beilei Zeng, Chuanyu You.

**Writing – review & editing:** Qilin Wang, Beilei Zeng, Yan Gui.
